# Research trends and hotspots in sclerotherapy for vascular malformations: bibliometric and visual analyses

**DOI:** 10.3389/fphys.2026.1861596

**Published:** 2026-06-11

**Authors:** Shanshan Li, Yanwen Liu, Chenggang Liu

**Affiliations:** 1 School of Basic Medical Sciences, Heilongjiang University of Chinese Medicine, Harbin, China; 2 Nanjing University of Aeronautics and Astronautics, Nanjing, Jiangsu, China

**Keywords:** bibliometric analysis, data visualization, research trends, sclerotherapy, vascular malformations

## Abstract

**Introduction:**

Vascular malformations are a group of congenital vascular developmental anomalies. According to the International Society for the Study of Vascular Anomalies classification, they are mainly divided into Slow-Flow and Fast-Flow lesions. Slow-Flow lesions are represented by venous malformations, lymphatic malformations, and capillary malformations; Fast-Flow lesions primarily include arteriovenous malformations and arteriovenous fistulae. As a minimally invasive interventional approach, sclerotherapy has become a first-line treatment for Slow-Flow vascular malformations; significant advances in techniques, agents, and combination therapies have been made in recent years. However, a systematic bibliometric analysis of overall research trends, knowledge structure, and frontier hotspots in this field is lacking.

**Methods:**

Using the Web of Science Core Collection and PubMed databases, we retrieved literature on sclerotherapy for vascular malformations from 2005 to 2025, including 2,040 and 1,855 articles, respectively. VOSviewer, CiteSpace and Bibliometrix were employed to analyze publication trends, collaboration networks, journal distribution, highly cited literature, keyword clustering, and burst detection. Cross-validation across the two databases was performed to enhance robustness.

**Results:**

Annual publications accelerated from 2015 and showed an explosive increase after 2020. China, the United States, and Japan were the top three publishing countries, with 535, 451, and 106 publications, respectively; the United States led in total citations. Shanghai Jiao Tong University, Shandong University, and Sungkyunkwan University were the most active institutions. Journal distribution followed Bradford’s law, with Zone 1 comprising 30 journals. Three-field (three-map) analysis revealed that core authors such as Lee BB and Do YS formed dense networks with key terms and journals. LDA topic modeling identified 15 topics; evolutionary analysis indicated a field shift from traditional sclerosants toward targeted therapies, electrochemical treatments, and artificial intelligence. Burst-term analysis confirmed sirolimus and intralesional bleomycin as sustained frontiers. PubMed and WoS results were highly consistent.

**Discussion:**

This study provides the first systematic 20-year knowledge map of the field of sclerotherapy for vascular malformations. China is the most productive country, though average per-article impact remains to be improved. The field is transitioning toward targeted therapies, physicochemical synergistic approaches, and multidisciplinary comprehensive management. Future work should strengthen combined targeted-drug and sclerotherapy approaches, promote AI-assisted precision treatment, and conduct high-quality prospective clinical studies.

## Graphical Abstract

**Figure:**
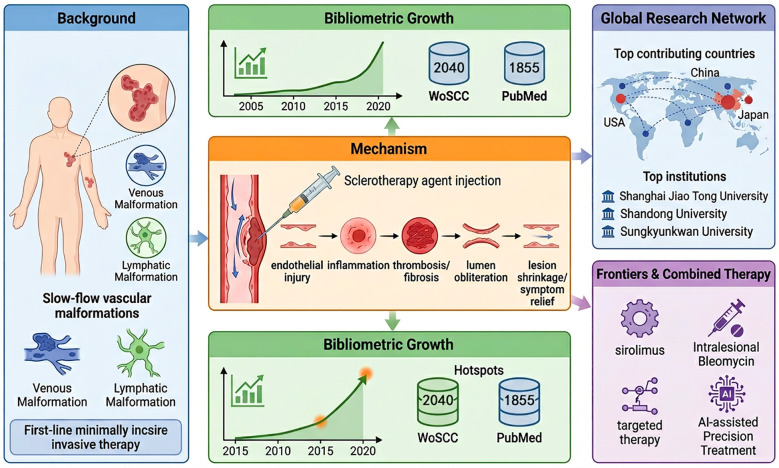

## 1 Introduction

Vascular malformations are congenital anomalies of blood or lymphatic vessels. According to the International Society for the Study of Vascular Anomalies (ISSVA) classification, they are divided into Slow−Flow and Fast−Flow malformations. Slow−Flow malformations primarily include venous malformations, lymphatic malformations, capillary malformations, and mixed malformations composed of these types; fast−flow malformations mainly comprise arteriovenous malformations and arteriovenous fistulas (Wassef et al., 2015; International Society for the Study of Vascular Anomalies, 2025). Unlike infantile hemangiomas, vascular malformations do not involute spontaneously; they typically grow proportionally with body development and may exhibit rapid progression or symptom exacerbation during puberty, pregnancy, or after trauma, persisting lifelong (Mulliken and Glowacki, 1982). Clinical manifestations vary with subtype, location, and lesion size: mild cases may be asymptomatic, whereas severe cases can cause pain, functional impairment, or be life−threatening (Liu et al., 2025). Conventional treatments include surgical excision, sclerotherapy, laser therapy, and symptomatic pharmacotherapy (Raja et al., 2024). However, extensive and complex lesions remain difficult to cure. Surgery is applicable only to focal lesions or for debulking; for diffuse lesions or those in anatomically challenging regions, complete resection is often impossible and recurrence rates are high (Markovic et al., 2020). Sclerotherapy, as a minimally invasive and repeatable interventional approach, has become the first−line treatment for Slow−Flow vascular malformations (particularly venous malformations and macro−cystic lymphatic malformations) (Leal et al., 2023). Recent advances in sclerotherapy include development of composite sclerosant formulations that enhance safety while maintaining efficacy (Liu et al., 2024). Nevertheless, for diffuse or multifocal disease the primary goals of sclerotherapy are symptom control, functional improvement, and enhanced quality of life rather than radiologic cure; thus recurrence and multiple treatment sessions are common, often requiring multidisciplinary management combining surgery or targeted medications (Shiraishi et al., 2026).

Bibliometrics applies mathematical and statistical methods to quantitatively and qualitatively analyze scientific literature, objectively revealing a field’s knowledge structure, developmental trajectory, and research frontiers (Hassan and Duarte, 2024). Although bibliometric analyses have been conducted on venous/lymphatic malformations overall, systematic studies specifically focused on sclerotherapy—the core therapeutic modality—are lacking. This study retrieved literature on sclerotherapy for vascular malformations from the Web of Science Core Collection and PubMed for 2005–2025 and performed bibliometric and visualization analyses using VOSviewer, CiteSpace and Bibliometrix, aiming to systematically map publication trends, key contributors, knowledge bases, research hotspots, and emerging directions to provide evidence for clinical practice and future research.

## 2 Methodology

### 2.1 Data sources and search strategies

**Figure 1:**
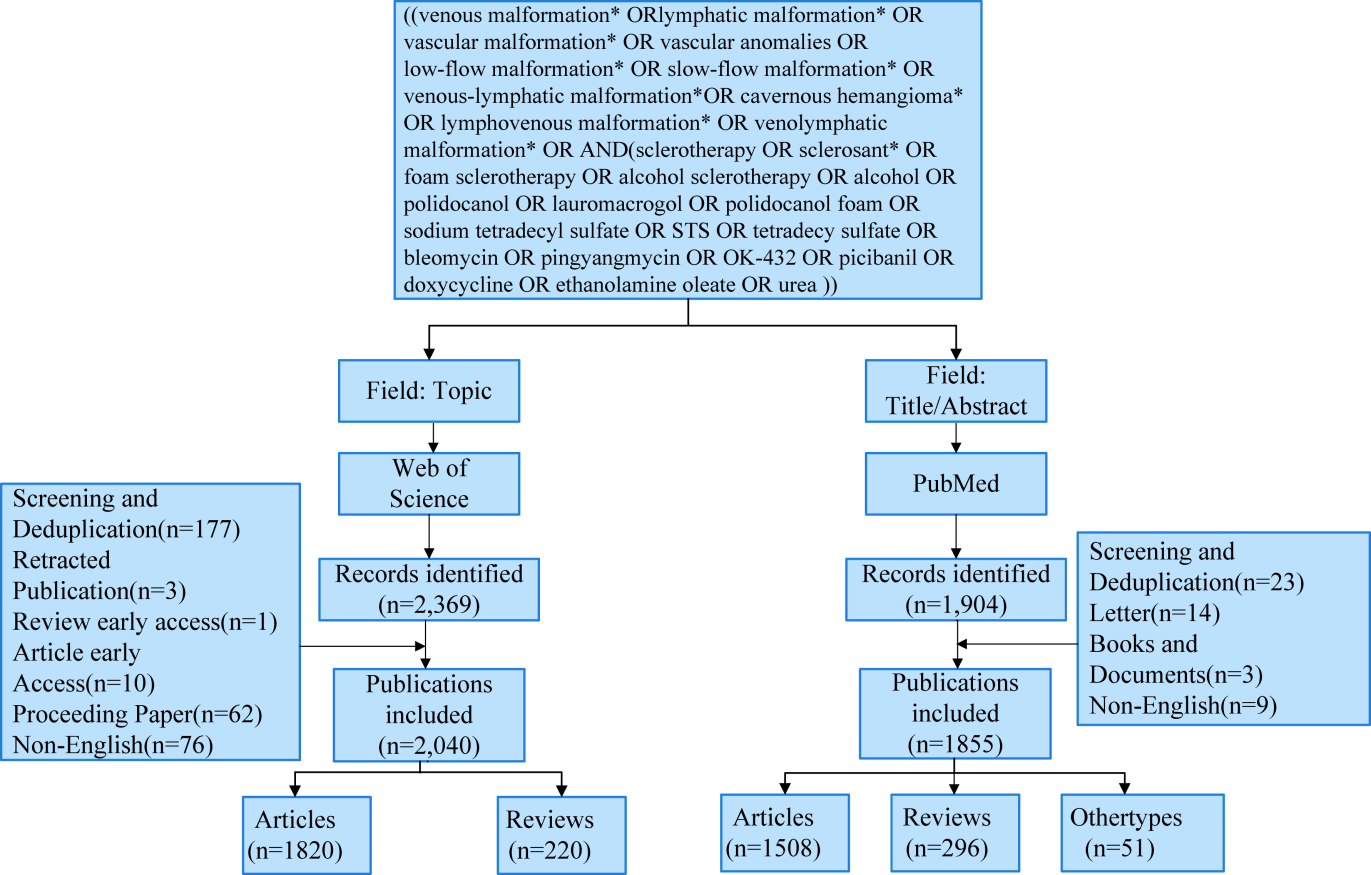
Workflow of the data search strategy.

This study retrieves literature from two independent databases: the Web of Science Core Collection (WoSCC) and PubMed. The Science Citation Index Expanded (SCI-Expanded) within WoSCC serves as the primary data source for bibliometric mapping and visual analysis, as it covers high-quality journals and its export format is compatible with VOSviewer and CiteSpace. PubMed is used as a supplementary data source for cross-database consistency analysis of publication trends, as well as supplementary analysis of journals, authors, countries, and institutions. The retrieval date is April 7, 2026, covering the time span from January 1, 2005, to December 31, 2025. The search strategy is constructed around keywords related to the treatment of vascular malformation sclerosis, using a combination of subject headings and free-text terms. The specific search formula in WoSCC is shown in **Figure 1**.The literature types are limited to Articles and Reviews, and the language is restricted to English. After deduplication, title and abstract screening, and full-text screening, a total of 2,040 WoSCC literature items were included. The parallel analysis in PubMed employs the same core search formula, with language restricted to English, yielding a total of 1,855 literatures after the same screening process. The complete retrieval strategy for WoSCC and PubMed, along with detailed inclusion and exclusion criteria, can be found in the **Supplementary Materials**.

### 2.2 Bibliometric analysis

We employed three bibliometric software tools for data analysis and visualization. The analysis process included data cleaning, descriptive statistics, collaboration network analysis, co-occurrence and co-citation analysis, emergence detection, and visualization presentation. Before network analysis, the author and institution names were standardized; the thesaurus function of VOSviewer was used to merge different name variants of the same institution into a standard form, and author disambiguation was completed by cross-checking the full author names, affiliated institutions, and accessible ORCID identifiers. The process for handling authors with the same name is detailed in the **Supplementary Materials**. VOSviewer (version 1.6.20) was used to construct collaboration networks of countries, institutions, and authors, as well as keyword co-occurrence networks and co-citation networks of journals and references. Association strength was used as the normalization method. Node size represents publication volume or citation frequency, line thickness represents association strength, and color represents different groups identified by clustering algorithms. The network layout was optimized using the Force Atlas2 algorithm. Complete parameter settings can be found in the **Supplementary Materials**. CiteSpace (version 6.4.R1) was used for emergence detection of keywords and references, generating keyword cluster timelines, and overlaying journal dual maps. The emergence detection algorithm was utilized to identify emerging research frontiers, while the time zone view was used to display the trajectory of thematic evolution. Complete parameter settings can be found in the **Supplementary Materials**. Topic modeling using latent Dirichlet allocation (LDA) was conducted using the topicmodels package in R. The number of topics was determined through grid search, selecting optimal parameters based on a combination of perplexity, topic coherence, stability, and interpretability. Model stability was verified by running with different random seeds, and topic interpretation was independently completed by two authors who discussed to reach consensus. The complete results of the grid search, model verification parameters, and interpretation processes can be found in the **Supplementary Materials**. Bibliometrix (R package, version 4.3.3) and its web interface Biblioshiny were used for descriptive statistics, Bradford’s law verification, three-field plot drawing, and thematic evolution analysis. The annual growth rate calculation employed an exponential growth model.

## 3 Results

### 3.1 Publication trends

According to the retrieval strategy described above, this study included 2,040 publications published between 2005 and 2025, comprising 1,748 Articles and 219 Reviews. The total number of references was 42,122, and the average citations per paper were 21.87. Basic information on research into sclerotherapy for vascular malformations is presented in **Table 1**.

**Table 1:** Summary of Basic Bibliographic Information on Sclerotherapy for Vascular Malformations.

Description	Results
Main information	
Timespan	2005:2025
Sources (Journals, Books, etc)	735
Documents	2040
Average citations per document	21.87
References	42122
Keyword	
Keyword Plus(ID)	3718
Author	
Authors	8597
Co-authors per document	6.04
International co-authorships%	13.91
Document types	
Article	1748
article; early access	10
article; proceedings paper	62
Article; retracted publication	3
Review	219
Review; early access	1

### 3.2 Contributions of countries to global publications

**Figure 2:**
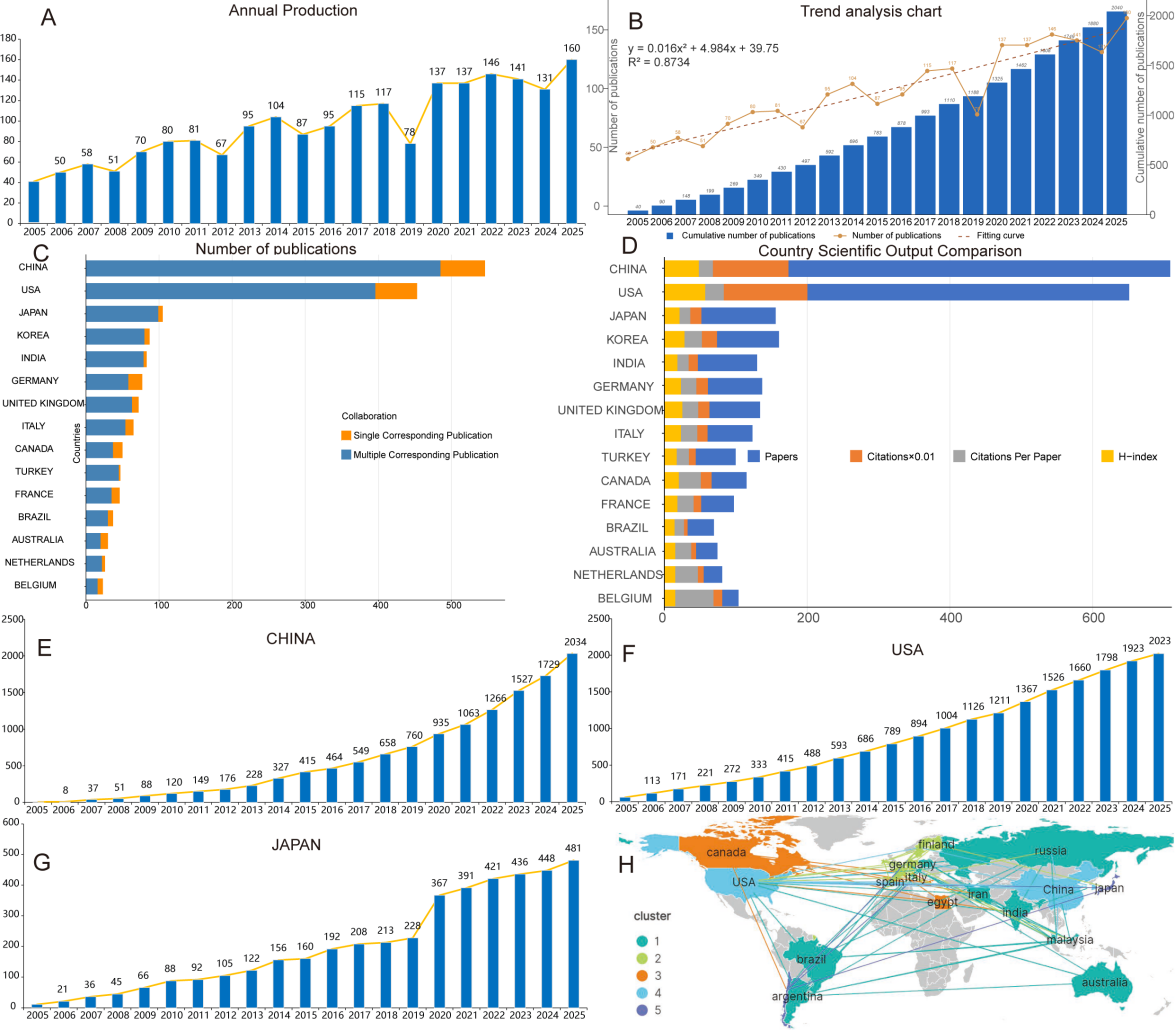
Publication trends and international cooperation in vascular malformations treatment research. **(A)** Global annual publication trend; **(B)** Cumulative publication volume and polynomial fitting curve (y = 0.016x² + 4.984x + 39.75, R² = 0.8734); **(C)** Distribution of publication volume by major countries; **(D)** Distribution of citation influence by country (total citation frequency vs average number of citations); **(E)** Cumulative publication trend in China; **(F)** Cumulative publication trend in the United States; **(G)** Cumulative publication trend in Japan; **(H)** National scientific research cooperation network map.

The annual publication trend for research on sclerotherapy for vascular malformations is shown in **Figure 2A**. From 2005 to 2014, the number of publications increased slowly (40 to 104 papers per year); from 2015 to 2018, the field entered an accelerated growth phase (rising from 87 to 117 papers); after a brief decline in 2019, publication numbers have grown explosively since 2020, reaching a peak of 160 papers in 2025. Based on the fitted model of cumulative publication counts in **Figure 2B**, research output in this field is expected to continue steady growth in the coming years.

By corresponding author country (**Figure 2C**), China produced the most publications over the past two decades (535 papers, 26.2%), followed by the United States (451 papers, 22.1%), Japan (106 papers, 5.2%), South Korea (87 papers, 4.3%), and India (83 papers, 4.1%). In terms of total citations, the United States ranked first, reflecting higher average impact per paper; China, Japan, Germany, and the United Kingdom followed (**Figure 2D**). Notably, the number of new publications from the United States, China, and Japan increased throughout the study period, with particularly marked growth after 2020 (**Figures 2E–G**). The international collaboration network is shown in **Figure 2H**. Five clusters were identified, with prominent Western European–American clusters centered on the United States and Germany and an Asia–Pacific cluster centered on China and Japan, although collaboration intensity between China and the United States was limited.

### 3.3 Distribution of publishing institutions

**Figure 3:**
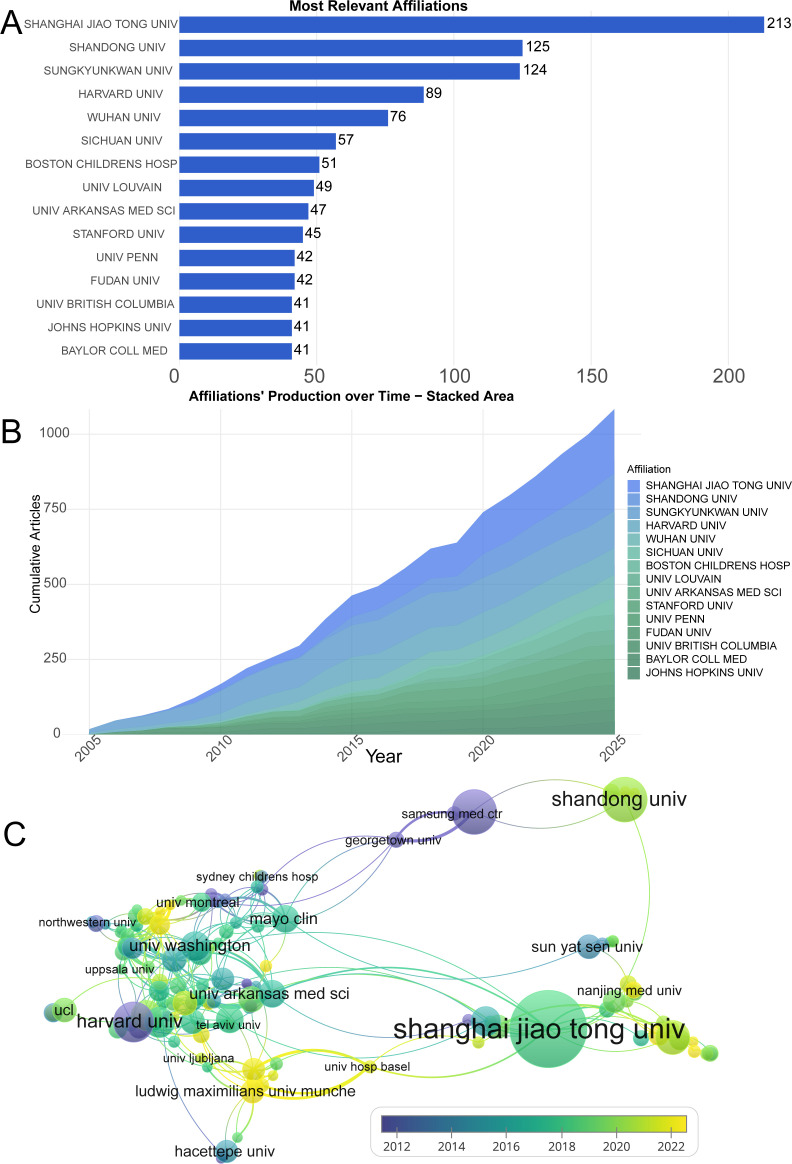
Distribution and cooperation network of major research institutions. **(A)** Top 15 institutions by publishing papers; **(B)** Time evolution trend of the top 15 institutions publishing papers; **(C)** Institutional cooperation network map.

Among the top 15 institutions by publication count, Shanghai Jiao Tong University ranks first, followed by Shandong University. Five are from China, seven from the United States, and the remainder from South Korea, Belgium, Canada, and others. Shanghai Jiao Tong University in China exhibited the most rapid growth, reaching 213 papers in 2025 to take first place; Shandong University in China significantly accelerated after 2015, accumulating 125 papers; Sungkyunkwan University in South Korea led early but slowed later, accumulating 124 papers; Harvard University in the United States started publishing early but has shown weak growth in recent years, accumulating 89 papers; the remaining institutions each accumulated between 40 and 80 papers (**Figure 3A**). **Figure 3B** presents the cumulative publication trends of the top 15 institutions: Chinese institutions experienced strong growth after 2015 and have established a leading advantage; although there are many U.S. institutions, most have slowed in later growth; European and Canadian institutions have also maintained steady output.

In the analysis of institutional collaboration networks, 14 clusters were identified (**Figure 3C**). Institutions such as Harvard University, Boston Children’s Hospital, and Harvard Medical School form a core U.S. research cluster, collaborating closely with the University of Pennsylvania, Johns Hopkins University, the University of Washington, and others. KU Leuven, University College London, the University of Amsterdam, and Ludwig Maximilian University of Munich form a European research cluster. Shanghai Jiao Tong University, Shandong University, Fudan University, the Chinese Academy of Medical Sciences, Sungkyunkwan University, and Seoul National University comprise an East Asian research cluster, within which Shanghai Jiao Tong University maintains relatively close collaborative ties with domestic Chinese institutions and Sungkyunkwan University in South Korea.

### 3.4 Distribution of authors’ publications

Consistent with Lotka’s law, authors publishing six or more papers account for less than 0.1%. As shown in **Table 2**, 20 authors published 20 or more papers. WANG Y from Guang’anmen Hospital, China, is the most prolific author in the field, with 40 publications. WANG H from Guilin University, China, has the highest h-index, indicating strong citation impact; closely following is DO YS from Sungkyunkwan University, South Korea. Notably, among the top 20 most productive authors, 15 are from China, three from South Korea, and two from Germany, reflecting the dominant role of Chinese researchers in this field.

**Table 2:** Ranking of prolific authors.

Author	Articles	Country	H-index	Affilliations	Publication year started
WANG Y	40	CHINA	17	Guang’anmen Hospital Jinan Hospital	2007
YANG X	36	CHINA	11	Shanghai Jiao Tong University	2014
LI J	33	CHINA	15	Shandong University, Jinan	2009
CHEN H	30	CHINA	12	Shanghai Jiaotong University	2009
LI X	30	CHINA	14	Chongqing Medical University	2013
ZHANG J	30	CHINA	15	Chongqing Medical University	2008
WANG L	26	CHINA	11	Sichuan University	2007
DO YS	25	KOREA	18	Sungkyunkwan University School of Medicine	2005
LIN X	25	CHINA	10	Shanghai Jiao Tong University	2008
LIU Y	25	CHINA	13	Guizhou University	2008
ZHANG X	25	CHINA	11	Children’s Hospital Affiliated to Shandong University	2015
WANG	24	CHINA	11	Qingdao University	2010
WANG H	24	CHINA	35	Guilin University	2010
WOHLGEMUTH WA	24	GERMANY	10	University Hospital Halle	2015
CHEN Y	21	CHINA	9	Southern Medical University	2010
FAN X	20	CHINA	12	China-Japan Friendship Hospital	2010
KIM DI	20	KOREA	17	Yonsei University	2005
KIM YW	20	KOREA	17	Hanyang University	2005
WILDGRUBER M	20	GERMANY	9	Regensburg University Medical Center	2017
ZHANG L	20	CHINA	14	Weifang No. 2 People’s Hospital,	2007

**Figure 4:**
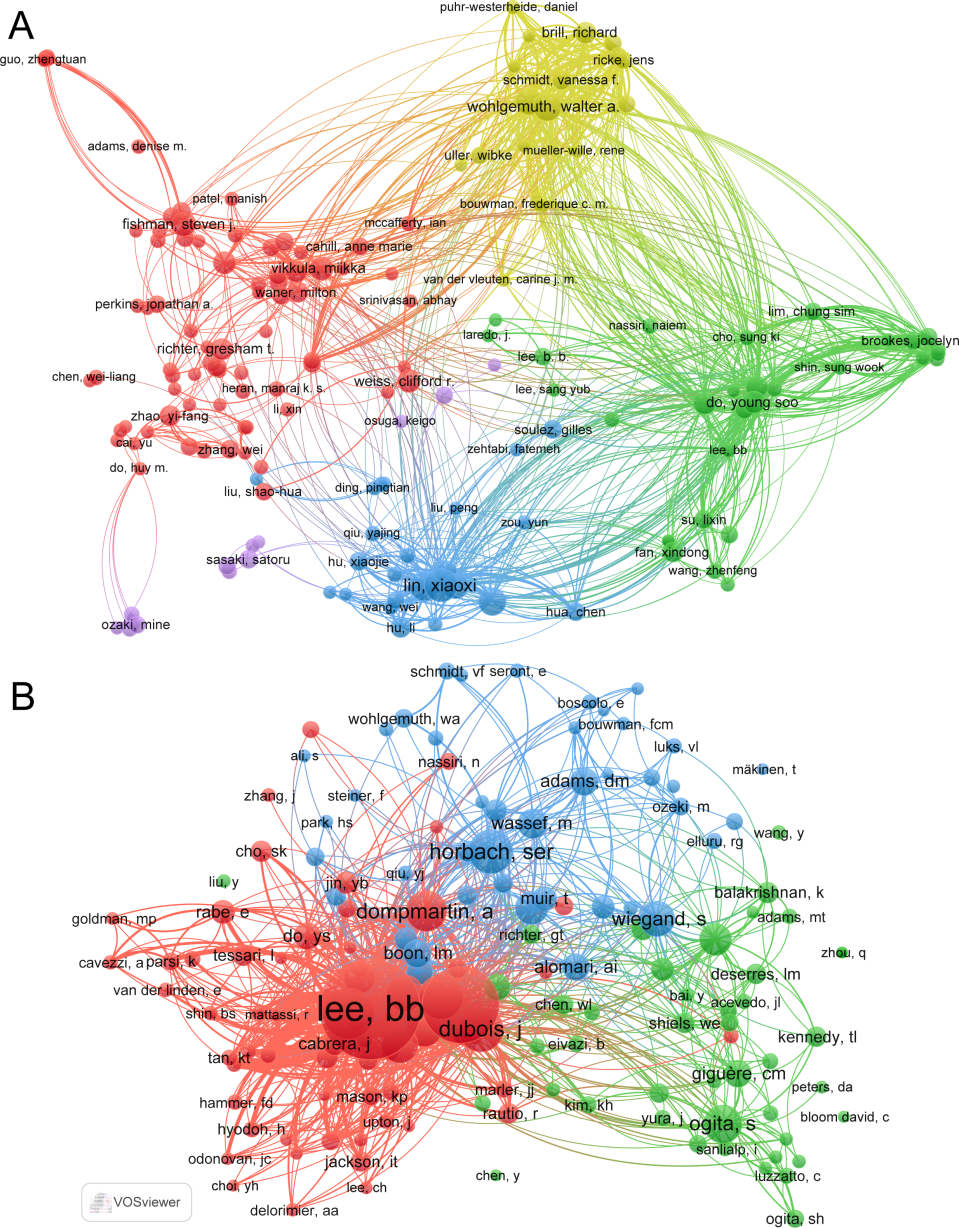
Author collaboration and co-citation network. **(A)** Author co-occurrence network (authors with ≥ 5 articles); **(B)** Author co-citation network (authors with ≥ 40 citations).

**Figure 4A** shows the citation network of authors in the field of sclerotherapy for vascular malformations. We identified 161 authors with at least five publications; these authors form five clusters (distinguished by color). Lee BB (green cluster), Lin Xiaoxi (center of the blue cluster), and Wohlgemuth WA (yellow cluster) occupy key nodes in the network, indicating their work is widely cited by subsequent researchers. Authors such as Fishman SJ, Vikkula M, and Richter GT show tight citation linkages within the red cluster. Do YS, as one of the core nodes of the green cluster, together with Lee BB, constitutes an important research direction in the field, reflecting the growing influence of Chinese and Korean researchers.

**Figure 4B** presents the co-citation network of authors in the field of sclerotherapy for vascular malformations. We identified 150 co-cited authors with at least 40 citations; these authors form three clusters (distinguished by color). Lee BB and Boon LM serve as core nodes of the red cluster and have active co-citation relationships with authors such as Dompmartin A and Richter GT. Adams DM, Ozeki M, Wohlgemuth WA, and Seront E exhibit close co-citation ties in the blue cluster, forming another group focused on sclerotherapy techniques and clinical outcome studies. Wiegand S, Balakrishnan K, and others form an independent research group within the green cluster.

### 3.5 Journal distribution

The journal distribution analysis shows that literature in this field follows a typical concentration–dispersion pattern, consistent with Bradford’s law describing core, related, and peripheral zones (**Supplementary Figure 1**). The core Zone 1 comprises 30 journals, which published about one-third of the papers(**Supplementary Table 1**). The top 20 journals by publication volume are listed in **Table 3**; JOURNAL OF PEDIATRIC SURGERY and PHLEBOLOGY are tied for highest (57 articles each), followed by CARDIOVASCULAR AND INTERVENTIONAL RADIOLOGY (49 articles) and JOURNAL OF VASCULAR AND INTERVENTIONAL RADIOLOGY (47 articles). High-impact journals such as CHEMICAL ENGINEERING JOURNAL (IF = 13.2, Q1) and JOURNAL OF MEMBRANE SCIENCE (IF = 9.0, Q1) also contain related publications, though with lower article counts. A three-field analysis linking authors, keywords, and journals shows that core authors, high-frequency terms, and principal publishing journals form a tightly integrated knowledge-production network (**Supplementary Figure 2**), reflecting the main structure and disciplinary boundaries of knowledge production in this field.

**Table 3:** Ranking of major publishing journals.

Journal name	H-index	TC	NP	Journal citation reports quartile	Impact factor
CARDIOVASCULAR AND INTERVENTIONAL RADIOLOGY	19	894	49	Q2	2.9
JOURNAL OF PEDIATRIC SURGERY	19	1248	57	Q1	2.4
JOURNAL OF VASCULAR AND INTERVENTIONAL RADIOLOGY	18	976	47	Q2	2.6
PHLEBOLOGY	18	1238	57	Q3	1.6
JOURNAL OF MEMBRANE SCIENCE	17	1164	18	Q1	9.0
INTERNATIONAL JOURNAL OF PEDIATRIC OTORHINOLARYNGOLOGY	16	691	38	Q4	1.3
JOURNAL OF VASCULAR SURGERY	15	803	18	Q2	3.6
JOURNAL OF VASCULAR SURGERY-VENOUS AND LYMPHATIC DISORDERS	14	478	37	Q3	3.0
DERMATOLOGIC SURGERY	13	517	32	Q3	2.2
JOURNAL OF ORAL AND MAXILLOFACIAL SURGERY	13	529	21	Q4	0.4
JOURNAL OF CRANIOFACIAL SURGERY	12	460	51	Q3	1.0
LARYNGOSCOPE	12	492	18	Q3	2.0
OTOLARYNGOLOGY-HEAD AND NECK SURGERY	12	680	14	Q3	2.5
CHEMICAL ENGINEERING JOURNAL	11	1044	11	Q1	13.2
EUROPEAN JOURNAL OF VASCULAR AND ENDOVASCULAR SURGERY	11	1046	15	Q1	6.8
AMERICAN JOURNAL OF ROENTGENOLOGY	10	261	12	Q1	6.1
BRITISH JOURNAL OF ORAL & MAXILLOFACIAL SURGERY	10	320	14	Q3	1.9
EUROPEAN RADIOLOGY	10	346	12	Q2	4.7
PEDIATRIC SURGERY INTERNATIONAL	10	289	18	Q3	1.6
PLOS ONE	10	418	14	Q3	2.6

Regarding citation influence, the United States has the highest total citations (11,715), followed by China (10,666) and South Korea (2,118). However, Singapore has the highest citations per article (60.2), followed by Belgium (53.5) and Austria (42.4), indicating that small but highly specialized research teams may produce work of outstanding impact. Canada (30.3) and the Netherlands (31.7) also have relatively high citations per article, reflecting the quality of their research (**Table 4**).

**Table 4:** National scientific research output and influence ranking.

Country	Articles	TC	Average article citations
USA	1536	11715	26
CHINA	1314	10666	19.9
KOREA	242	2118	24.3
GERMANY	295	1629	21.4
JAPAN	368	1608	15.2
UNITED KINGDOM	196	1526	21.8
CANADA	180	1483	30.3
ITALY	232	1439	22.8
INDIA	174	1318	15.7
BELGIUM	117	1231	53.5
FRANCE	216	1056	23
TURKEY	152	961	17.2
NETHERLANDS	94	823	31.7
AUSTRALIA	106	720	22.5
SINGAPORE	22	542	60.2
BRAZIL	113	495	13.4
IRAN	57	474	20.6
SPAIN	79	459	20
EGYPT	31	356	27.4
AUSTRIA	106	297	42.4

The top 20 locally cited papers in the field of sclerotherapy for vascular malformations are shown in **Table 5**. The most locally cited paper is Horbach SER (2016) in Journal of Plastic, Reconstructive & Aesthetic Surgery, with 108 local citations and 166 global citations; this paper systematically reviewed the efficacy of sclerotherapy for venous malformations. Other highly cited works include Alomari AI (2006) on percutaneous sclerotherapy and Mathur NN (2005) on OK−432 treatment for lymphatic malformations. Notably, several early studies from 2005 to 2010 (e.g., Do YS 2005, Legiehn GM 2008, Dompmartin A 2010) constitute the knowledge base of modern sclerotherapy, covering technical innovations, complication management, and clinical outcome assessment.

**Table 5:** Ranking of highly cited literature in the field.

Document	DOI	Year	Local citations (LC)	Global citations (GC)
HORBACH SER, 2016, J PLAST RECONSTR AES	10.1016/j.bjps.2015.10.045	2016	108	166
ALOMARI AI, 2006, J VASC INTERV RADIOL	10.1097/01.RVI.0000239104.78390.E5	2006	83	123
MATHUR NN, 2005, INT J PEDIATR OTORHI	10.1016/j.ijporl.2004.08.008	2005	81	113
LEGIEHN GM, 2008, RADIOL CLIN N AM	10.1016/j.rcl.2008.02.008	2008	77	195
DO YS, 2005, RADIOLOGY	10.1148/radiol.2352040449	2005	75	170
DOMPMARTIN A, 2010, PHLEBOLOGY	10.1258/phleb.2009.009041	2010	72	214
VAN DER VLEUTEN CJM, 2014, CARDIOVASC INTER RAD	10.1007/s00270-013-0764-2	2014	72	101
PERKINS JA, 2010, OTOLARYNG HEAD NECK	10.1016/j.otohns.2010.02.026	2010	71	161
SMITH MC, 2009, LARYNGOSCOPE	10.1002/lary.20041	2009	70	113
HORBACH SER, 2016, PLAST RECONSTR SURG	10.1097/PRS.0000000000001924	2016	68	102
PUIG S, 2005, EUR J RADIOL	10.1016/j.ejrad.2004.07.023	2005	65	125
NEHRA D, 2008, J PEDIATR SURG	10.1016/j.jpedsurg.2007.10.009	2008	65	87
CHO SK, 2006, J ENDOVASC THER	10.1583/05-1769.1	2006	63	205
YAMAKI T, 2008, J VASC SURG	10.1016/j.jvs.2007.11.026	2008	62	113
BEHRAVESH S, 2016, CARDIOVASC DIAGN THE	10.21037/cdt.2016.11.10	2016	62	165
CHAUDRY G, 2014, CARDIOVASC INTER RAD	10.1007/s00270-014-0932-z	2014	61	92
OKAZAKI T, 2007, J PEDIATR SURG	10.1016/j.jpedsurg.2006.10.012	2007	60	156
CAHILL AM, 2011, J PEDIATR SURG	10.1016/j.jpedsurg.2011.07.004	2011	59	95
ELLURU RG, 2014, SEMIN PEDIATR SURG	10.1053/j.sempedsurg.2014.07.002	2014	57	179
ACEVEDO JL, 2008, OTOLARYNG HEAD NECK	10.1016/j.otohns.2007.11.018	2008	55	108

### 3.6 Keywords and hotspots

**Figure 5:**
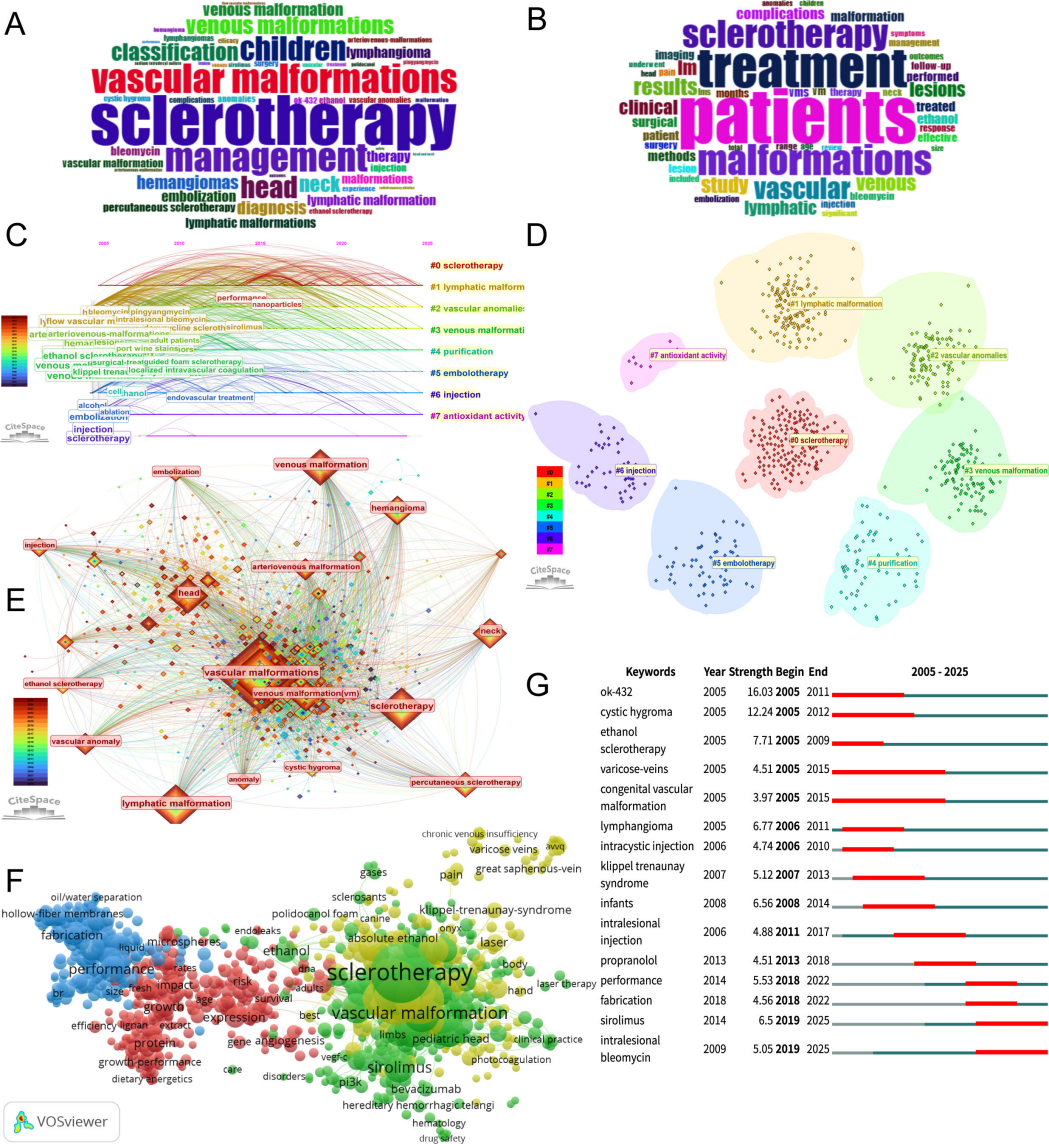
Keyword visualization analysis in the field of vascular malformations and sclerosis treatment. **(A)** Keyword cloud; **(B)** Title word cloud; **(C)** Keyword clustering timeline view; **(D)** Keyword clustering map; **(E)** Keyword co-occurrence network overall view; **(F)** Keyword co-occurrence network clustering view; **(G)** Keyword emergence analysis.

According to Web of Science subject categories, research on sclerotherapy for vascular malformations is primarily distributed across radiology, nuclear medicine and medical imaging, surgery, pediatrics, and peripheral vascular disease. The high-frequency keyword word cloud (**Figure 5A**) shows “venous malformation,” “sclerotherapy,” “bleomycin,” “lymphatic malformation,” and “embolization” as core terms. The title word cloud (**Figure 5B**) further highlights research foci such as “treatment,” “complications,” and “imaging.” Keyword clustering analysis identified seven major clusters with good cluster network quality (**Figures 5C–E**). Thematic evolution analysis indicates the field is shifting from empirical technical operations toward systematic comprehensive management, with continuous maturation of core themes (**Supplementary Figure 3**).

Latent Dirichlet Allocation (LDA) topic modeling identified 15 research topics encompassing four major dimensions: treatment techniques and methods, efficacy assessment and safety, basic and translational research, and clinical comprehensive management (**Supplementary Table 2**). Topic evolution shows early studies focused on fundamental techniques such as cell activity and efficacy assessment and CT-guided interventions; in recent years, patient benefit and complication management, artificial intelligence, and combined treatment with anhydrous ethanol sclerotherapy/embolization have shown upward trends, corroborating the field’s transition from single-technique operations to integrated management models.

Burst term analysis (**Figure 5G**) reveals early hotspots centered on traditional sclerosants, while recent sustained bursts for sirolimus and intralesional bleomycin indicate that targeted therapy and intralesional bleomycin have become frontier directions. The keyword co-occurrence network (**Figure 5F**) further confirms the central role of sclerosants and targeted therapies. Overall, research hotspots in this field have shifted from traditional sclerosants toward targeted therapies, interventional radiology techniques, and electrochemical treatments, with interdisciplinary approaches such as artificial intelligence beginning to attract attention.

### 3.7 Comprehensive analysis of PubMed

**Figure 6:**
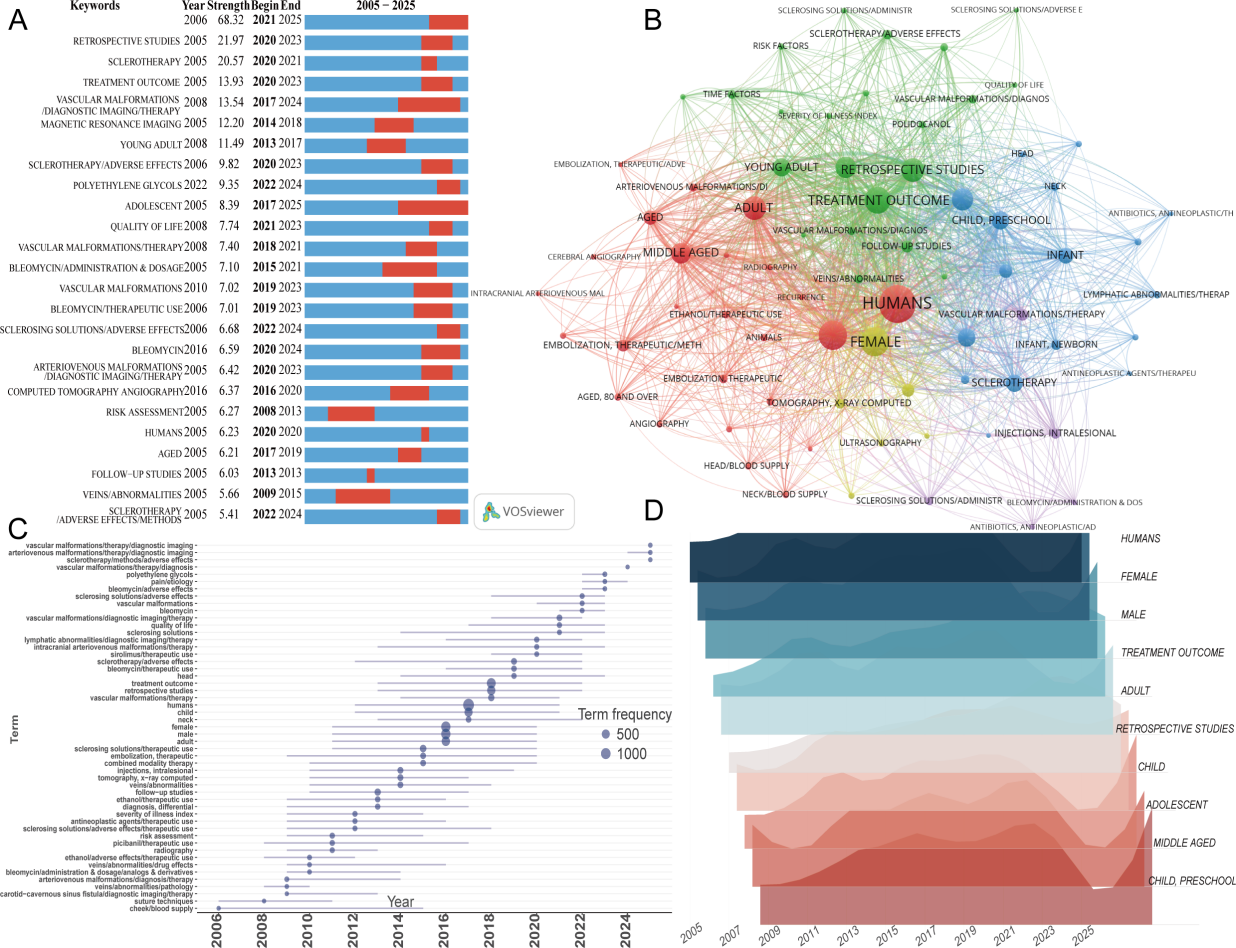
Keyword analysis of PubMed database. **(A)** Keyword emergence detection; **(B)** Keyword co-occurrence network; **(C)** Time evolution trend of high-frequency subject keywords; **(D)** Timeline chart of dynamic evolution of keywords.

The PubMed database included 1,855 articles from 582 journals, involving 7,073 authors (**Table 6**), a scale comparable to the Web of Science Core Collection. Burst-word detection and temporal trend analysis (**Figures 6A, D**) revealed the dynamic evolution of research hotspots: early burst terms included veins/abnormalities (2009–2015) and sclerotherapy/adverse effects (2020–2023), reflecting initial attention to identification of vascular abnormalities and treatment safety; between 2015 and 2020, bleomycin/therapeutic use, magnetic resonance imaging, and quality of life successively emerged, indicating widespread application of bleomycin, the popularization of imaging assessment, and the rise of clinical outcome evaluation; since 2021, retrospective studies (strength 68.32), polyethylene glycols, and sclerosing solutions/adverse effects have become the strongest burst terms, suggesting dominance of retrospective studies, emergence of new sclerosing agents, and continued focus on adverse event monitoring. These trends corroborate a shift in the field from “technical procedures” toward “comprehensive management,” with greater attention to patient quality of life and long-term follow-up.

**Table 6:** Summary of basic information of PubMed database.

Description	Results
Main information	
Timespan	2005:2025
Sources (Journals, Books, etc)	582
Documents	1855
Keyword	
Keyword Plus(ID)	3784
Author	
Authors	7073
Co-authors per document	5.68
International co-authorships%	6.038
Document types	
Article	1508
Review	296
Other types	51

Trend topic analysis (**Figure 6C**) further shows that imaging assessment, treatment outcomes, and studies of special populations have been frequently appearing in recent years. The keyword co-occurrence network (**Figure 6B**) demonstrates that sclerotherapy, vascular malformations, bleomycin, ethanol, polidocanol, magnetic resonance imaging, treatment outcome, and quality of life constitute the research backbone, forming a tightly connected network around three major themes: sclerosing agent application, image-guided procedures, and efficacy evaluation. These core nodes align closely with the burst-term analysis, jointly outlining the field’s knowledge structure as it evolves from traditional sclerosing agents toward targeted therapies and comprehensive management.

**Figure 7:**
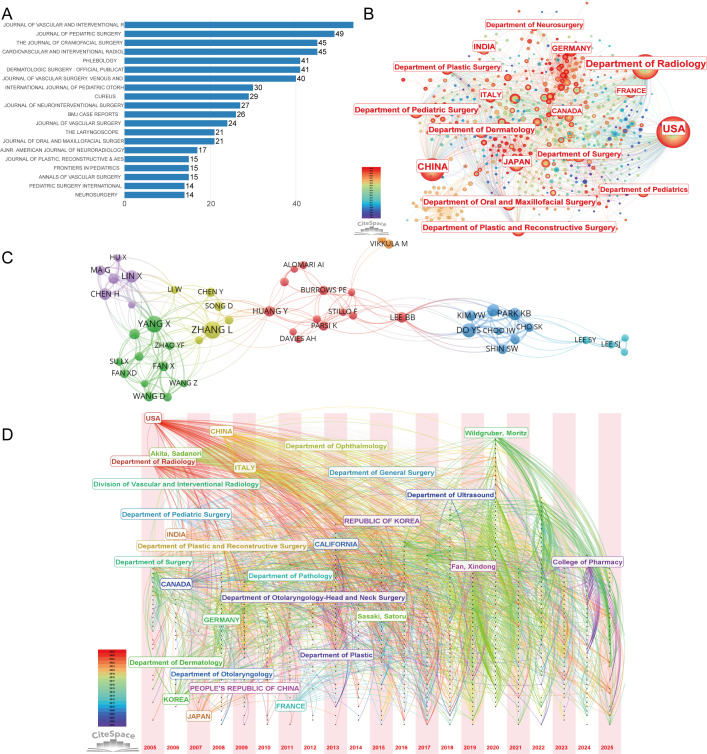
PubMed database validation analysis. **(A)** Distribution of major journals; **(B)** Author co-occurrence network; **(C)** National-institutional cooperation network; **(D)** Distribution of subject areas.

In order to assess the consistency of the research results across different databases, a parallel analysis was conducted on the PubMed database. Journal distribution (**Figure 7A**) shows the top five publishing journals are JOURNAL OF PEDIATRIC SURGERY, JOURNAL OF VASCULAR AND INTERVENTIONAL RADIOLOGY, CARDIOVASCULAR AND INTERVENTIONAL RADIOLOGY, PHLEBOLOGY, and DERMATOLOGIC SURGERY, largely consistent with the WoS ranking. In the author co-occurrence network (**Figure 7B**), Vikkula M, Alomari AI, Burrows PE, Lee BB, and Do YS are core nodes, highly overlapping with WoS results. The country/region collaboration network (**Figure 7C**) indicates the United States, Germany, Italy, Canada, Japan, and India as primary nodes, forming two major collaborative circles in Europe–North America and the Asia–Pacific region, with limited direct US–China collaboration. Discipline distribution (**Figure 7D**) shows otorhinolaryngology, plastic and reconstructive surgery, radiology, and pediatric surgery as the main participating departments, reflecting multidisciplinary collaboration. In summary, PubMed and WoS exhibit a high degree of consistency in journal rankings, core authors, collaboration patterns, and disciplinary distribution, indicating that the main bibliometric findings have good robustness across databases.

## 4 Discussion

This study is the first to perform a systematic bibliometric and visualization analysis in the field of sclerotherapy for vascular malformations, integrating the Web of Science Core Collection and PubMed databases to reveal the overall knowledge structure, evolution of research hotspots, and status of clinical research in this field. Publication trends show that the field entered an accelerated growth phase beginning in 2015 and has experienced explosive growth since 2020, indicating that sclerotherapy, as a first-line technique for Slow-Flow vascular malformations, is receiving increasing academic attention. According to ISSVA classification, Slow-Flow types include venous and lymphatic malformations, which—due to slow blood flow and fragile vessel walls—are the primary indications for sclerotherapy; Fast-Flow types, namely arteriovenous malformations, require combined embolization or surgery. In pediatric vascular malformations, genetic mosaicism and TEK and PIK3CA mutations causing vessel wall defects explain at the molecular level the lesions’ sensitivity to sclerosants.

In terms of publication volume, China has surpassed the United States to become the global leader, representing a shift from the traditional perception of Western dominance. This change may be attributed to China’s policy support for promoting minimally invasive interventional techniques, abundant case resources, and the rapid dissemination of sclerotherapy as an technique-dependent. However, China’s citations per article remain lower than those of countries such as the United States, Singapore, and Belgium, suggesting that while quantity has grown rapidly, there remains room to improve research quality. Small but highly specialized research teams from countries like Singapore and Belgium produce work with higher per-article impact, a phenomenon worth noting.

At the institutional level, Chinese institutions such as Shanghai Jiao Tong University and Shandong University have shown particularly rapid growth since 2015, while U.S. institutions, although numerous, have slowed in later years, indicating a shift of the research center toward Asia. Author analysis shows that Chinese researchers dominate among high-output authors, but high productivity and high citation impact do not completely overlap, indicating that influence is not simply positively correlated with output quantity. Regarding journal distribution, the JOURNAL OF PEDIATRIC SURGERY and PHLEBOLOGY are the most prolific journals, reflecting the central roles of pediatric and venous disease specialties; involvement of materials science journals reflects the field’s interdisciplinary nature, as sclerotherapy depends not only on clinical technique but also benefits from development of novel sclerosants such as foams and nanomaterials.

The evolution of research hotspots clearly traces the field’s shift from technical procedures toward integrated management. Burst keyword analysis shows that early hotspots concentrated on traditional sclerosing agents such as OK−432 and ethanol. Bleomycin, owing to its strong endothelial-sclerosing effect, was widely used in macrocystic lymphatic malformations. Absolute ethanol, because of its potent endothelial-destructive capability, was applied to refractory venous malformations, but severe complications such as nerve injury limited its use. In recent years, development of combination sclerosing formulations has become an important trend; for example, bleomycin combined with polidocanol foam (BPF) has maintained efficacy comparable to traditional absolute ethanol regimens while markedly reducing the number of treatments and showing a clear safety advantage with no observed severe nerve injury. After 2019, sirolimus and endoluminal (intralesional) bleomycin injection have remained persistent burst keywords. The rise of sirolimus aligns with the increasing use of mTOR inhibitors in complex vascular anomalies; multiple clinical trials have confirmed that sirolimus can effectively relieve pain and improve function, though it is difficult to eradicate lesions (Seront et al., 2023; Wang et al., 2025). At the same time, endoluminal injection of bleomycin, because of its safety profile superior to ethanol, has become a first−line choice for venous and lymphatic malformations.

It is worth noting that keyword clustering analysis identifies electrochemotherapy as an independent cluster, and burst detection also confirms its continued active status in recent years. This bibliometric signal indicates that physical-chemical synergy therapies, represented by bleomycin electrochemotherapy (BEST), are receiving increasing academic attention. Preliminary studies have explored the mechanism of BEST, which significantly enhances the intracellular uptake of bleomycin by momentarily increasing cell membrane permeability through reversible electroporation, thereby achieving efficient endothelial cell apoptosis at low drug doses (Schmidt et al., 2024). A prospective study and multicenter data (n=89) show that BEST can reduce the median volume of lesions by 82.3% with only 1 to 2 treatments (Goldann et al., 2025). The BEST standardized operating procedures published in 2024 provide clinical guidelines for this technique (Muir et al., 2024). Large-volume lesions (>1000 cm³) may require multiple BEST treatments, and temporary skin hyperpigmentation (6 out of 30 cases) was the main adverse reaction, with no severe complications such as pulmonary fibrosis reported (Haehl et al., 2025). This evidence supports BEST as a first-line choice for refractory venous malformations rather than merely a salvage therapy after traditional treatment failure. Therefore, based on the current bibliometric signal and existing clinical evidence, the BEST technique marks a shift in sclerotherapy from purely chemical destruction to a physical-chemical synergy model, representing one of the most noteworthy advances in this field in recent years.

Meanwhile, the keyword clustering analysis of this research identifies artificial neural network as an independent cross-technical cluster. From a bibliometric perspective, this indicates that artificial intelligence has begun to penetrate this field. Existing proof-of-concept studies have explored preliminary applications of AI in several directions. First, lesion automatic segmentation and quantification. Accurately quantifying the volume changes of irregular lesions is the objective basis for evaluating therapeutic efficacy. Studies have used the 3D U-Net deep learning model for the automatic segmentation of MRI images of craniofacial venous malformations, achieving a Dice similarity coefficient of 60.62% in the test set, demonstrating the feasibility of automated lesion delineation. Another recent study explored segmentation methods based on unsupervised generative models, providing a new technical path to address the challenge of scarce labeled data for rare diseases, with the best model achieving a Dice coefficient of 0.50 ± 0.03 in animal experiments (Ryu et al., 2022) (Fraissenon et al., 2026). Second, treatment response prediction. The need for multiple treatment sessions in sclerotherapy makes predicting individual efficacy of significant clinical value. A concept validation prospective study, by analyzing the radiomic characteristics of diffusion-weighted MRI images before and after sclerotherapy in patients with venous malformations, preliminarily demonstrated the potential of this technology to predict improvements in patients’ quality of life after treatment, providing early evidence-based support for imaging-driven treatment response prediction (Gerwing et al., 2022). Overall, the application of AI in the sclerotherapy of vascular malformations is still in its initial stages, with current evidence mainly consisting of single-center small sample proof-of-concept studies, and the generalizability of the models still needs to be validated. Based on the current bibliometric trends and existing proof-of-concept results, as multi-center high-quality imaging databases are established and algorithms optimized, AI may have translational potential in precise lesion quantification, personalized efficacy prediction, and clinical decision support, but this requires validation through more high-quality research.

In the field of targeted therapy, bibliometric analysis shows that sirolimus has surged to become one of the most frequently mentioned therapeutic keywords and has been identified as a sustained frontier through burst detection. This bibliometric signal aligns with the increasingly widespread clinical application of mTOR inhibitors in complex vascular malformations in recent years (Hori et al., 2020). According to existing clinical evidence, sirolimus, by inhibiting the PI3K-AKT-mTOR signaling pathway, has become the first targeted drug for the treatment of vascular malformations. However, sirolimus acts on the downstream mTOR kinase of the PI3K-AKT-mTOR pathway and lacks high specificity for inhibiting various upstream mutations. Directly targeting the upstream PI3Kα, Alpelisib, has resulted in an average lesion volume reduction of 53% in the PIK3CA mutant cohort, significantly outperforming the 21% in the TEK mutant cohort, demonstrating the advantages of genotype-driven precision therapy, with relevant confirmatory clinical trials currently ongoing (Li et al., 2025). Based on the aforementioned bibliometric trends and clinical evidence, it is reasonable to speculate that future genetic testing prior to sclerosis treatment may become one of the exploratory directions for individualized treatment plans. Furthermore, new generation PI3Kα inhibitors, such as RLY-2608 and LOXO-783, are currently under development to provide treatment options for complex vascular malformations with central nervous system involvement (Zheng et al., 2025). For diffuse or multifocal lesions, studies have suggested that sirolimus could serve as a systemic treatment method, complementing local sclerotherapy (Adams et al., 2016). Its dosing strategy is being optimized, with low-target concentration regimens reducing the incidence of adverse events while maintaining efficacy. Clinical trials of local sirolimus formulations targeting superficial microcystic lymphatic malformations are evaluating their potential to reduce systemic side effects (Borst, 2024). If these strategies are confirmed, the combined application of sirolimus and sclerotherapy will be more feasible (Dodds et al., 2020). More cutting-edge is a study in 2024 demonstrating the efficacy of the KRAS G12C inhibitor sotorasib in relevant vascular malformations, suggesting that this field may be entering a stage of exploration driven by genotype-based precision therapy (Fraissenon et al., 2024). The TIE2 signaling axis, as a specific target for venous malformations with TEK mutations, is gaining attention; Rebastinib has successfully treated a case of progressive facial venous malformation, providing an alternative for TEK mutations where sirolimus is ineffective (Triana and Lopez-Gutierrez, 2023). In summary, from the perspective of bibliometric trends and existing clinical evidence, combining sclerotherapy and targeted drugs to form a comprehensive plan of local sclerotherapy plus systemic targeting is a research direction worth further exploration.

Based on the current research and the findings from the bibliometric analysis of this study, as well as existing clinical evidence, five development directions that warrant ongoing tracking can be preliminarily identified: ①Standardization of physical-chemical synergistic technologies; ② Exploration of genotype-driven personalized therapies (i.e., selecting Alpelisib for PIK3CA mutations, Rebastinib for TEK mutations, and Sotorasib for KRAS mutations); ③ Development of local targeted drug delivery systems; ④ Feasibility studies on AI-assisted precision medicine; ⑤ Innovation in clinical trial methodologies for rare diseases. It should be emphasized that the aforementioned directions are preliminary summaries based on bibliometric trend analysis and existing clinical evidence, and their clinical translational feasibility and priority require further research validation.

The analysis of international cooperation networks shows that a close cooperation circle has formed among European and American countries, while the Asia-Pacific region is constituted by another cooperation circle that includes China, Japan, and Australia. The direct cooperation link strength between China and the United States is relatively limited, indicating a need to strengthen trans-Pacific scientific collaboration in the future. As the two countries with the highest volume of publications, China’s and the United States’ international cooperation proportion is quite low, which may be related to their large domestic research scale, but it also suggests that there is still room for improvement in both countries in utilizing international resources and promoting collaborative innovation. However, it should be noted that the volume of publications does not directly equate to scientific leadership or global influence; quality indicators such as average citations per paper, the proportion of international cooperation, and the contribution of highly cited literature are equally important. The field of rare diseases, in particular, requires international cooperation to accumulate cases and share experiences. To evaluate the cross-database consistency of the WoS analysis results, we conducted a parallel comparative analysis with PubMed, and the results showed that the two databases were highly consistent in terms of journal ranking, author co-occurrence networks, national cooperation patterns, and institutional distribution, further supporting the trend of transitioning from technical operations to comprehensive management in this field.

## 5 Limitations

There are the following limitations in this study: First, only English literature was included, which may have missed significant research published in other languages, resulting in language bias; Second, bibliometric analysis has inherent lagging characteristics, and literature published in the past two years may be underestimated in its impact due to insufficient citation time accumulation; Third, in the cluster analysis, certain terms may be weakly associated with themes, possibly due to interdisciplinary noise; Fourth, there may be a small number of authors with the same name not fully distinguished or variants of names not completely merged in the author collaboration network, and the conclusions are mainly based on macro patterns; Fifth, the clinical prevalence of genotype-driven therapy is limited, and some targeted drugs are still in the experimental stage, with long-term data yet to be accumulated; Sixth, bibliometric analysis cannot exclude the impact of self-citation on citation metrics, as self-citation behavior by authors or journals may affect the objectivity of citation frequency to some extent; Seventh, there are inherent differences in the coverage, subject areas, and types of literature between WoSCC and PubMed. Although the cross-database comparison results of this study show a high degree of consistency, the differences between the two databases may still have a slight impact on individual indicators. Additionally, LDA topic modeling is based on word co-occurrence, and the interpretation of topic labels has a certain degree of subjectivity.

## 6 Conclusion

This study systematically depicts the research landscape of sclerotherapy for vascular malformations from 2005 to 2025. China has become the most productive country in this field, with institutions such as Shanghai Jiao Tong University leading development. Research hotspots have shifted from traditional sclerosing agents toward targeted therapies, electrochemical treatments, and applications of artificial intelligence. Future efforts should strengthen combined studies of targeted drugs and sclerotherapy, promote the intelligent development of interventional radiology techniques, and conduct multicenter prospective clinical studies to validate the long-term efficacy of emerging therapies.

## Data Availability

The original contributions presented in the study are included in the article/**Supplementary Material**. Further inquiries can be directed to the corresponding author/s.
