# Supplementary figures and images for: Research trends and hotspots in sclerotherapy for vascular malformations: bibliometric and visual analyses
Source: Front Physiol. 2026 Jun 11;17:1861596. doi: 10.3389/fphys.2026.1861596 (PMC13293944; doi:10.3389/fphys.2026.1861596)

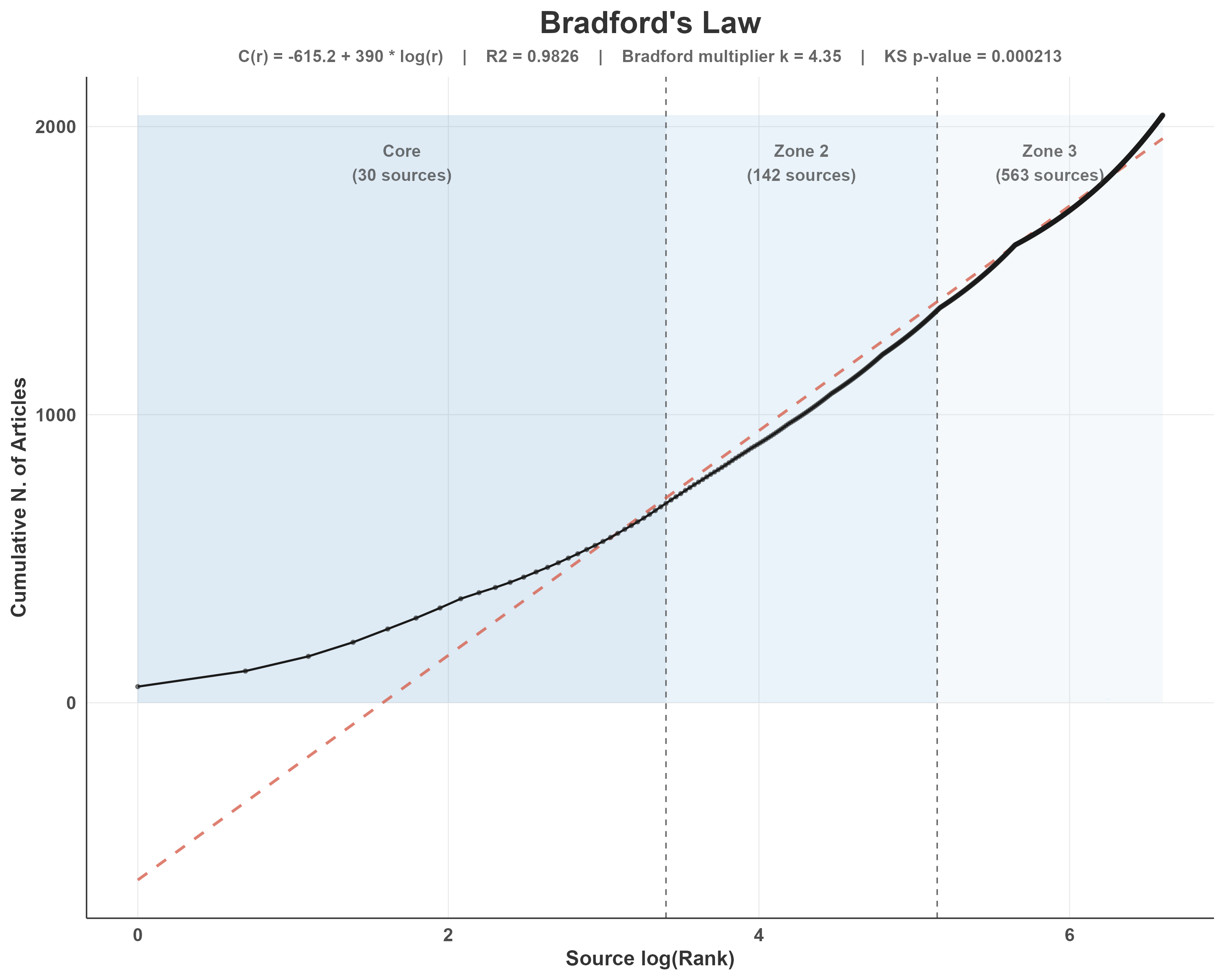

Supplement: Supplementary file 1 [file Image1.tif]

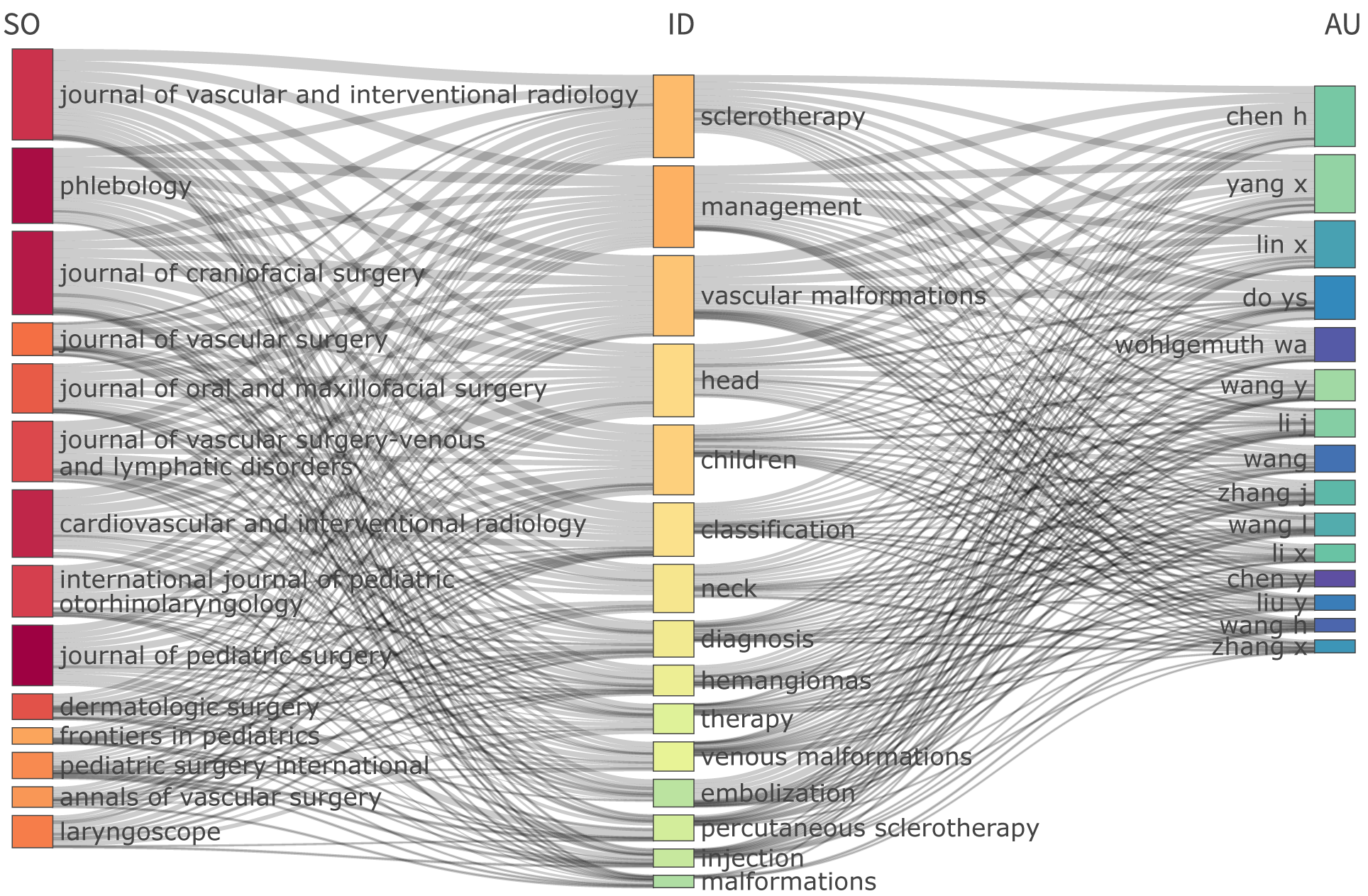

Supplement: Supplementary file 2 [file Image2.tif]

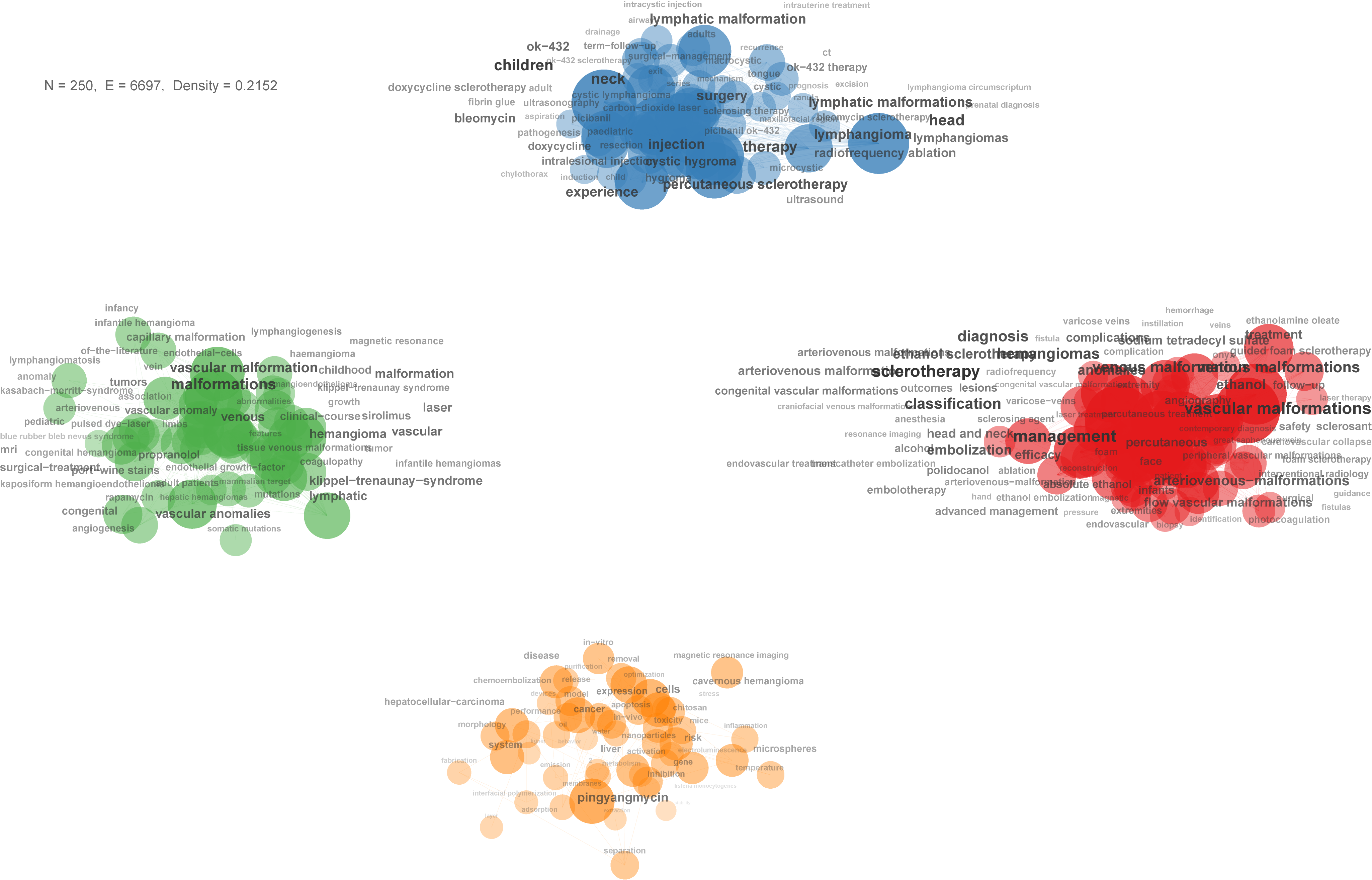

Supplement: Supplementary file 3 [file Image3.tif]
